# Functional Validation of Different Alternative Splicing Variants of the *Chrysanthemum lavandulifolium ClNUM1* Gene in Tobacco

**DOI:** 10.3390/cimb46060314

**Published:** 2024-05-25

**Authors:** Wenxin Zhang, Hai Wang, Yuning Guo, Xueying Hao, Yanxi Li, Wenting He, Xiang Zhao, Shiyi Cai, Xuebin Song

**Affiliations:** College of Landscape Architecture and Forestry, Qingdao Agricultural University, Qingdao 266109, China; 20212210009@stu.qau.edu.cn (W.Z.); wh672831@126.com (H.W.); 20212110007@stu.qau.edu.cn (Y.G.); 20212210002@stu.qau.edu.cn (X.H.); lyx33091@126.com (Y.L.); qauylhwt@163.com (W.H.); crazyz18@163.com (X.Z.); qaucaishiyi@163.com (S.C.)

**Keywords:** *C. lavandulifolium*, *ClNUM1*, alternative splicing, abiotic stress

## Abstract

The Asteraceae are widely distributed throughout the world, with diverse functions and large genomes. Many of these genes remain undiscovered and unstudied. In this study, we discovered a new gene *ClNUM1* in *Chrysanthemum lavandulifolium* and studied its function. In this study, bioinformatics, RT-qPCR, paraffin sectioning, and tobacco transgenics were utilized to bioinformatically analyze and functionally study the three variable splice variants of the unknown gene *ClNUM1* cloned from *C. lavandulifolium*. The results showed that *ClNUM1.1* and *ClNUM1.2* had selective 3′ splicing and selective 5′ splicing, and *ClNUM1.3* had selective 5′ splicing. When the corresponding transgenic tobacco plants were subjected to abiotic stress treatment, in the tobacco seedlings, the *ClNUM1.1* gene and the *ClNUM1.2* gene enhanced salt and low-temperature tolerance and the *ClNUM1.3* gene enhanced low-temperature tolerance; in mature tobacco plants, the *ClNUM1.1* gene was able to enhance salt and low-temperature tolerance, and the *ClNUM1.2* and *ClNUM1.3* genes were able to enhance low-temperature tolerance. In summary, there are differences in the functions of the different splice variants and the different seedling stages of transgenic tobacco, but all of them enhanced the resistance of tobacco to a certain extent. The analysis and functional characterization of the *ClNUM1* gene provided new potential genes and research directions for abiotic resistance breeding in *Chrysanthemum*.

## 1. Introduction

*Chrysanthemums* are traditional Chinese flowers and among the most important ornamental flowers in the world, with high ornamental and application value [1]; however, at the same time, *Chrysanthemums* are very susceptible to abiotic stresses, which greatly limits their application, and their genetic background is relatively complex, so progress in *Chrysanthemum* resistance research has been relatively slow [2]. *C. lavandulifolium* is a diploid plant of the genus *Chrysanthemum* in the family Asteraceae with a relatively simple genetic background and is also one of the important parents of modern *Chrysanthemum* species, making it a model plant for the study of plants in the family Asteraceae. Therefore, genetics studies in *C. lavandulifolium* are important for resistance breeding in *Chrysanthemum* [3].

Studies on the resistance of Chrysanthemum plants have been carried out in many aspects, such as heat resistance and insect resistance, and the main bases are NAC, MYB, and WRKY transcription factors family [4,5,6,7,8,9,10,11,12]. For example, the *SND1* and *NST1* genes in the NAC family regulate secondary cell wall and lignin synthesis, increasing the broad-spectrum resistance of *Leucanthemella linearis* [13]. *Chrysanthemum × morifolium CmWRKY15-1* enhanced resistance to chrysanthemum white rust by regulating the expression of *CmNPR1* [14]. *Chrysanthemum indicum* var.aromaticum *CiMYB32* responds to drought stress [15]. In the process of studying the *NST1* gene of *C. lavandulifolium*, we accidentally cloned the unknown gene *ClNUM1* and its alternative splicing and the gene has a conserved structural domain of Nuclease-associated DNA-binding domain 3 (NUMOD3).

NUMOD3 is a homing endonucleases and related proteins. Sitbon et al. identified four new short conserved sequence structural domains in homing endonucleases and related proteins [16]. NUMODs were named the new structural domains nuclear-associated modular DNA-binding structural domains. These domains are modular and occur in various combinations. One structural domain consists of a motif whose structure has been described as a new sequence-specific DNA-binding helix. Sequence similarity suggests that the other two structural domains are novel helix–turn–helix DNA-binding domains. Four families of homing endonucleases are known—HNH, GIY-YIG, His-Cys box, and LAGLIDADG—each characterized by and named after a short-conserved sequence motif in the structural domain of its nuclease [17]. Among them, NUMOD3 is found in single-stranded and tandem repeats of GIY-YIG and HNH proteins and includes the DNA-binding domain of the I-TevI homologous endonuclease [18,19]. It forms a unique extended structure that wraps around the DNA. This region binds to DNA in a sequence-specific manner, helically inserting and twisting the DNA groove [20].

Alternative splicing events, including alternative 5′ and 3′ splice site selection, exon skipping, and intron retention [21], are widespread mechanisms in eukaryotes [22]. In alternative splicing, a single gene produces different protein variants through different splice site combinations [23,24,25]. Selective splicing is involved in the regulation of various plant biological activities, including plant responses to biotic and abiotic stresses [26,27], regulation of plant flowering [28], and regulation of the plant biological clock [29]. Chaudhary et al. proposed that under stress conditions, plants buffer the level of normal protein synthesis by alternative splicing, reduce the translation of a large portion of the transcriptome, and produce protein isoforms that are adapted to the protein isoforms needed under stress [30]. Splice variants have been studied in plants such as maize, rice, citrus, and *Arabidopsis thaliana* [31,32,33,34], but there are still few studies on *Chrysanthemum*.

Since the conserved structural domain of these three splice variants is NUMOD3, we named these three splice variants *ClNUM1.1, ClNUM1.2*, and *ClNUM1.3*. The nucleotide sequences of the three variable splices were compared with the homologous gene *CsNST1* in the NCBI database, and the homology of the nucleotide sequences was found to be as high as 83%. Afterward, the three splice variants of this gene were transferred into tobacco, and the biological functions of the different variants in the model plant tobacco were identified via RT-qPCR, morphometric indexes, physiological indexes, and paraffin sections, to study the function of *ClNUM1* in plants under abiotic stress so as to explore potential functional genes for resistance breeding of *Chrysanthemum*.

## 2. Materials and Methods

### 2.1. Plant Materials, Vectors and Strains

The *C. lavandulifolium* histocultures and wild-type big-leaf tobacco (*Nicotiana tabacum* var. *macrophylla*), vector Super35S::GFP, *E. coli* strain DH5α, and Agrobacterium strain GV3101 used in this study were obtained from the group of Associate Prof. Xuebin Song at Qingdao Agricultural University.

### 2.2. Homologous Gene Cloning

*Cynara scolymus* is a plant in the family Asteraceae and is closely related to *C. lavandulifolium*. The coding region of the *CsNST1* gene found in the NCBI database (accession number LOC112517473, NCBI) was used as a template for primer design, and the primer sequences were as follows (Table 1):

Then, the gene was cloned using *C. lavandulifolium* as a template according to the instructions of 2 × Phanta Flash Master Mix (Dye Plus) (Vazyme Biotech, Nanjing, China), and the obtained products were subjected to agarose gel electrophoresis migration experiments and then recovered by gel recovery using a FastPure Gel DNA Extraction Mini Kit (Vazyme Biotech, Nanjing, China), and the obtained products were sent to the company for sequencing to obtain different splice variants of *ClNUM1* gene: *ClNUM1.1*, *ClNUM1.2* and *ClNUM1.3*.

### 2.3. Stress Treatment of Chrysanthemum lavandulifolium

We transplanted *C. lavandulifolium* seedlings of 5 cm in height and good growth condition from sterile planting bottles (1/2 MS + 30 g/L sucrose + 6 g/LAGAR) and planted them in an artificial climate chamber at 25 °C, 16 h of light, 8 h of darkness, 3000 lux of light and 70% air humidity. Different treatments were applied to *C. lavandulifolium* after one week of incubation. The plants exposed to salt treatment were watered with 200 mmol/L NaCl, the drought treatment simulates drought conditions by not watering for a specified period of time, and the plants exposed to low-temperature treatment were placed in an incubator at 4 °C and sampled at 0 h, 1 h, 4 h, 8 h, 12 h, 24 h, and 36 h. We carried out at least three replicates per treatment.

### 2.4. RNA Extraction and RT-qPCR

We carried out RT-qPCR using wild-type *C. lavandulifolium* under different treatments to verify the expression patterns of the three transcripts under stress conditions. A certain amount of *C. lavandulifolium* and tobacco tissues was quick-frozen in liquid nitrogen and then quickly ground into powder and transferred to RNase-free 2 mL centrifuge tubes. Then, total RNA was extracted according to the instructions for the FastPure Plant Total RNA Isolation Kit (Vazyme Biotech, Nanjing, China). cDNA synthesis was performed according to the instructions for the HiScript III RT SuperMix for qPCR (+gDNA wiper) kit (Vazyme Biotech, Nanjing, China). RT-qPCR was performed on a StepONE Plus system (Applied Biosystems, Waltham, MA, USA) using SYBR qPCR Master Mix (Vazyme Biotech, Nanjing, China) and specific primers. The data obtained were analyzed by the 2^−ΔΔCt^ method, and the internal reference gene was *Actin7* (Table 2).

### 2.5. Agrobacterium Transformation Method

Three splice variants were recombined with the overexpression vector Super35S::GFP using the homologous recombination kit from Vazyme (ClonExpress^®^ Ultra One Step Cloning Kit). The recombinant plasmids were transferred into the receptor cells of Agrobacterium tumefaciens GV3101. The transformed A. tumefaciens colonies were selected on LB-agar plates containing 50 mg L^−1^ kanamycin, 50 mg L^−1^ rifampicin, and 50 mg L^−1^ gentamicin. Positive colonies were picked for PCR amplification and identification, after which the colonies were subjected to the configuration of infiltration solution and transformed tobacco as described [35]. After screening and characterization, the transgenic tobaccos were obtained.

### 2.6. Transgenic Tobacco Resistance Screening and Management and Maintenance Methods

In this study, Super35S::GFP was used as the control group (CK), and Super35S::GFP, Super35S::*ClNUM1.1*, Super35S::*ClNUM1.2*, and Super35S::*ClNUM1.3* tobacco seeds were sown in screening medium (MS + 50 mg/L Hgy + 30 g/L sucrose + 6 g/L agar) and incubated at 25 °C with 16 h of light and 8 h of darkness and 3000 lux light intensity. After the growth of true leaves, tobacco seedlings with similar growth status were picked and transplanted to the stress treatment medium in an aseptic environment as well as to an artificial climate chamber for subsequent treatment experiments.

### 2.7. Abiotic Stress Treatments and Morphometric Measurements

#### 2.7.1. Stress Treatment of Transgenic Tobacco at the Seedling Stage

Tobacco seedlings with the same growth status in the screening medium were transferred to MS, MS + 200 mmol/L NaCl, and MS + 200 µmol/L ABA(Abscisic-Acid) media, ABA is used to simulate drought stress [13]. The incubation conditions of the control, salt, and ABA treatments were at 25 °C, 16 h of light and 8 h of darkness, and 3000 lux light intensity, and that of the low-temperature treatment was at 4 °C, 16 h of light and 8 h of darkness, and 3000 lux light intensity. After 24 days of treatment in the medium, the tobacco plants were carefully removed, and the roots were carefully cleaned from the medium. After cleaning, the control, salt, ABA, and low-temperature treated tobacco plants were subjected to fresh weight and root length determination (Table S1).

#### 2.7.2. Stress Treatment of Transgenic Tobacco at the Mature Stage

Tobacco seedlings with the same growth status in the screening medium were selected and transferred to a charcoal substrate for two to three weeks and were subjected to control, salt, drought, and low-temperature treatments when they reached maturity. The control treatments were watered every other week, the salt treatments were watered with 200 mmol/L NaCl every other week, the drought treatments were subjected to simulated drought conditions by artificially controlling the water content of the soil, and the low-temperature treatments were placed in a 4 °C incubator. The control, drought, and salt treatments were all incubated at 25 °C. After 15 days of treatment in the artificial climate chamber, the growth status of the tobacco was photographed, and the height of the stalks was measured. Afterward, the stress-treated tobacco was rehydrated, and the height of the stalks was measured and photographed after seven days (Table S2).

### 2.8. Measurement of Physiological Parameters

To better illustrate the function of the splice variants, the chlorophyll content was determined in control and treated tobacco seedlings grown in an artificial climate chamber. Leaf samples were rinsed with distilled water and blotted dry with filter paper. The sides of the main veins of the leaves were cut into filaments <1 mm in width, and a 0.2 g sample was weighed out. The leaves were then immersed in 25 mL of 95% ethanol and left in the dark for 3 days. Absorbance was measured at wavelengths of 470 mm, 649 mm, and 665 mm using a UV spectrophotometer (Hitachi; Tokyo, Japan), and, finally, the total chlorophyll content was calculated.

### 2.9. Paraffin Sectioning

We selected prime-aged tobacco with the same growth state and cut a 1.5 cm long part from 1/3 of the stem of the transgenic tobacco. The selected stalks were treated in FAA fixative (70% alcohol/formalin/acetic acid 18:1:1) for 24 h. The material was soaked in a mixture of hydrogen peroxide and glacial acetic acid (1:1) for 48 h to soften the material and then dehydrated with ethanol and embedded in paraffin wax. The samples were divided into 10 μm sections by a slicer. Afterward, fenugreek solid green staining was performed, and the thickness of the cell wall and the size of the cells were observed using a light microscope.

### 2.10. Statistical Analysis

All experiments were set up with three or more biological replicates. Data are expressed as the mean ± SD (standard deviation) and were analyzed by a *t*-test (* *p* ≤ 0.05 and ** *p* ≤ 0.01).

## 3. Results

### 3.1. Bioinformatics Analysis of the ClNUM1 Splice Variants

**Figure 1:**
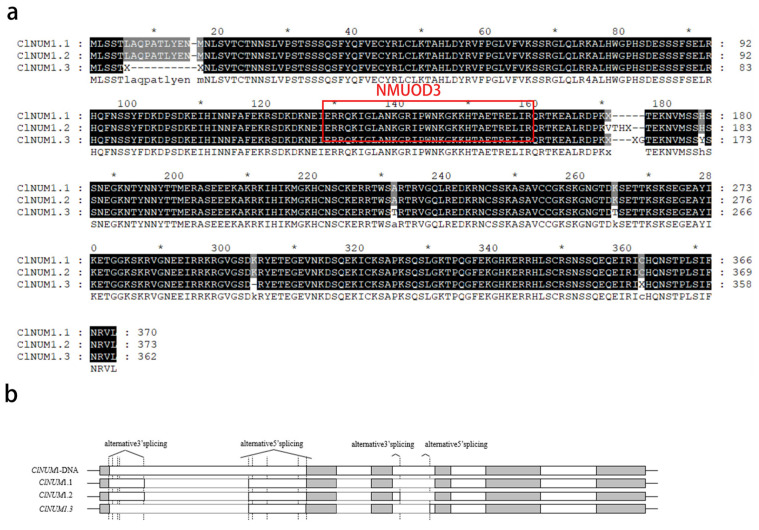
Bioinformatics analysis of three different splice variants. (**a**) Comparison of amino acid sequences. Black is the same part of the amino acid, gray is the different part of the amino acid. (* represents the length sequence number of the omitted amino acid.) (**b**) Schematic structure of *ClNUM1.1*, *ClNUM1.2*, and *ClNUM1.3*. The gray parts represent exons, and the white parts represent introns.

Three alternative splicing variants of *ClNUM1* were cloned from *C. lavandulifolium,* named *ClNUM1.1*, *ClNUM1.2*, and *ClNUM1.3* (Figure S1). *ClNUM1.1* was 1164 bp in total length and encoded 370 amino acids, while *ClNUM1.2* was 1176 bp and encoded 373 amino acids; *CLNUM1.3* was 1134 bp and encoded 362 amino acids (Figure 1a). *ClNUM1.1* and *ClNUM1.2* had both selective 3′ splicing and selective 5′ splicing, and *ClNUM1.3* had selective 5′ splicing (Figure 1b).

However, three variable splices of this gene were found in the cloning results. Alternative splicing generates multiple mRNA transcripts from a single gene by assembling exons differently using selective splice sites in precursor mRNAs.

Further analysis of the three variables found that the conserved structural domains of these three variable splices were different from those of the NST gene, and the three variable splices belonged to the conserved structural domain of NUMOD3 after comparison.

### 3.2. ClNUM1.1, ClNUM1.2, and ClNUM1.3 in C. lavandulifolium Show Different Responses to Stress Treatments

**Figure 2:**
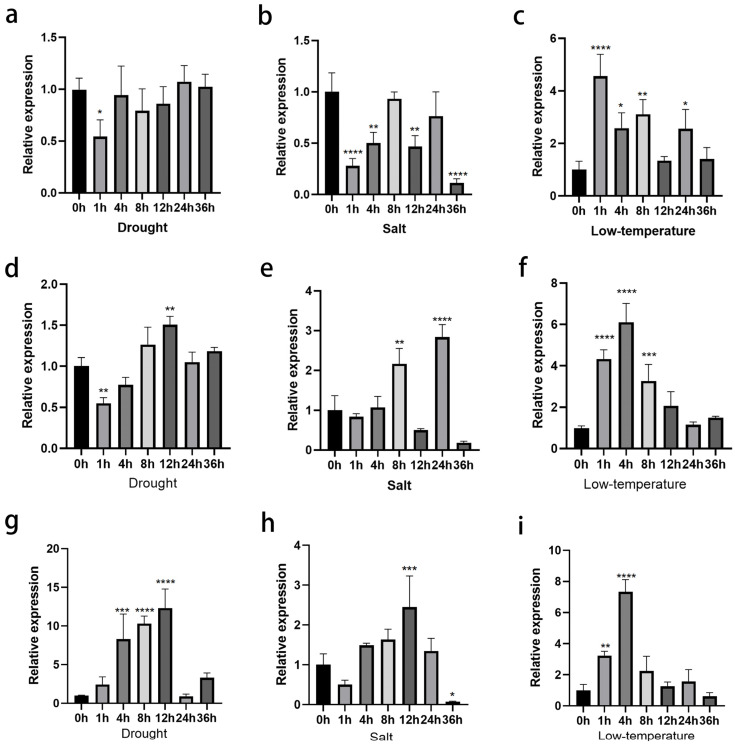
Expression levels of *ClNUM1.1*, *ClNUM1.2*, and *ClNUM1.3* in *C. lavandulifolium* under stress treatments. (**a**–**c**) Expression levels of *ClNUM1.1* in *C. lavandulifolium* under drought, salt, and low-temperature treatments. (**d**–**f**) Expression levels of *ClNUM1.2* in *C. lavandulifolium* under drought, salt, and low-temperature treatments. (**g**–**i**) Expression levels of *ClNUM1.3* in *C. lavandulifolium* under drought, salt, and low-temperature treatments. All experiments were set up with three or more biological replicates (independent sample *t*-test; * *p* < 0.05, ** *p* < 0.01, *** *p* < 0.001, **** *p* < 0.0001).

To further investigate the response of *ClNUM1.1*, *ClNUM1.2,* and *ClNUM1.3* in *C. lavandulifolium* under abiotic stress conditions, we comparatively analyzed the expression levels of the *ClNUM1.1*, *ClNUM1.2*, and *ClNUM1.3* genes in wild-type *C. lavandulifolium* under different stress treatments (Figure 2).

The expression of *ClNUM1.1* under drought and salt treatments was lower than that before the treatments, with a minimum decrease of 45.5% at 1 h in drought treatment and a minimum decrease of 88.7% at 36 h in salt treatment, while the expression of *ClNUM1.1* under low-temperature treatment showed an overall trend higher than that before the treatments and reached a peak at 1 h, with a 3.56-fold increase in expression. Under drought treatment, the expression of *ClNUM1.2* at 1 h and 12 h was significantly different from that before treatment, with a decrease of 45% and an increase of 50.7%, respectively. Under salt treatment, the expression increased significantly at 8 h and 24 h, with an increase of 1.16-fold and 1.84-fold, respectively, and the expression increased under low-temperature treatment compared with that before treatment, with increases of 3.34-fold, 5.13-fold, and 2.29-fold at 1 h, 4 h, and 8 h, respectively. The expression of *ClNUM1.3* under drought treatment was elevated compared with that before treatment, increasing 7.30-fold, 9.30-fold, and 11.33-fold at 4 h, 8 h, and 12 h. Under salt treatment the expression of *ClNUM1.3* was highest at 12 h, increasing 1.44-fold, and there was a significant increase in the expression of *ClNUM1.3* under low-temperature treatment at 1 h and 4 h, increasing 2.22-fold and 6.33-fold, respectively, compared with that before treatment.

These results clearly show that *ClNUM1.1*, *ClNUM1.2*, and *ClNUM1.3* in *C. lavandulifolium* all responded under the stress treatments, but the different splice variants responded differently to each treatment.

### 3.3. Phenotypic Analysis of Young ClNUM1 Transgenic Tobacco under Abiotic Stress

**Figure 3:**
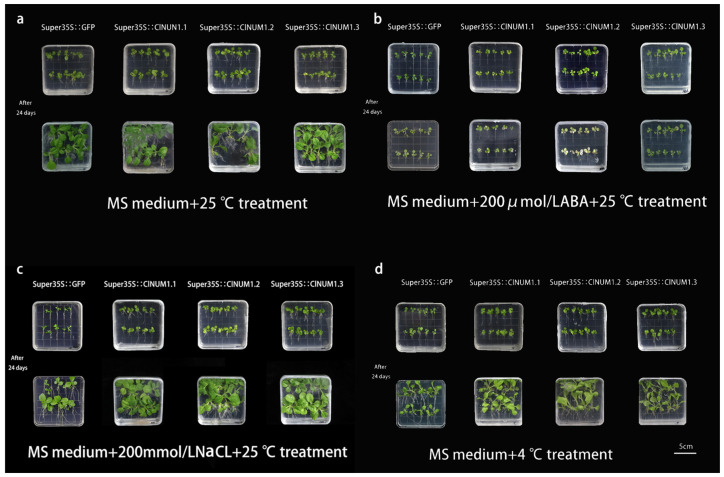
Growth status of tobacco under different treatments in the medium: (**a**) growth status under control treatment, (**b**) growth status under ABA treatment, (**c**) growth status under salt treatment, and (**d**) growth status under low-temperature treatment.

**Figure 4:**
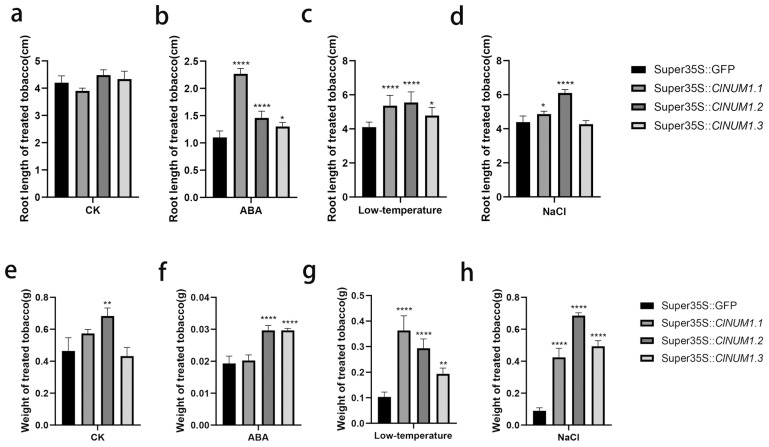
Root length and fresh weight of tobacco under different treatments after 24 days. (**a**–**d**) Root length of transgenic tobacco under control, drought, low-temperature, and salt treatments. (**e**–**h**) Fresh weight of transgenic tobacco under control, drought, low-temperature, and salt treatments All experiments were set up with three or more biological replicates (independent sample *t*-test; * *p* < 0.05, ** *p* < 0.01, **** *p* < 0.0001).

To investigate the function of the *ClNUM1.1*, *ClNUM1.2,* and *ClNUM1.3* splice variants, the present study was carried out to determine the root length, fresh weight, and chlorophyll content of the treated transgenic tobacco by abiotic stress treatment. After 24 days of treatment, we found that compared with before treatment, tobacco grew significantly under CK, NaCl, and low-temperature treatments, but the growth signs were not obvious under ABA conditions (Figure 3). The measurements showed that there was no significant difference in root length among the transgenic tobacco plants at 25 °C with 16 h of light and 8 h of darkness and 3000 lux, and the fresh weight of *ClNUM1.1* and *ClNUM1.3* was not significantly different from that of CK; the fresh weight of *ClNUM1.2* increased by 47.5% compared with that of CK; under ABA treatment, *ClNUM1.2* and *ClNUM1.3* showed an increase in root length and fresh weight compared to CK, their root length increased by 32.7% and 18.2%, respectively, and their fresh weight increased by 53.4%, showing a significant difference, while *ClNUM1.1* showed an increase in root length by 106.1% compared to CK, and its fresh weight was not significantly different; the root length and fresh weight of all three transgenic tobacco lines were increased under low-temperature treatment compared with CK, with a 30.6%, 35.2%, and 16.5% increase in root length and 251%, 183.8%, and 87.4% increase in fresh weight for *ClNUM1.1*, *ClNUM1.2,* and *ClNUM1.3*, respectively, showing significant differences; under salt treatment, the fresh weight of all three transgenic tobacco lines was increased and significantly different from CK, and the root length of *ClNUM1.1* and *ClNUM1.2* was increased by 10.8% and 39%, respectively, compared to that of CK, but the root length of *ClNUM1.3* showed little change compared to that of CK (Figure 4). We found that the growth of CK was inhibited under all the stress treatments, as well as the growth of the *ClNUM1.1*, *ClNUM1.2,* and *ClNUM1.3* transgenic tobacco lines under ABA treatment.

The experimental results showed that *ClNUM1.1* transgenic tobacco had a strong ability to tolerate salt and low temperature at the seedling stage; *ClNUM1.2* transgenic tobacco had a strong ability to tolerate salt and low temperature; and *ClNUM1.3* transgenic tobacco had a strong ability to tolerate low temperature.

### 3.4. Phenotypic Study of ClNUM1 Transgenic Tobacco at Maturity under Abiotic Stress and Determination of Physiological Indexes

To further illustrate the function of the splice variants under abiotic stress treatments, we observed the growth status of mature transgenic tobacco seedlings under stress treatments and statistically compiled the changes in the stem height of tobacco before and after rehydration under the stress treatments, as well as the chlorophyll content of the transgenic tobacco after the treatments.

**Figure 5:**
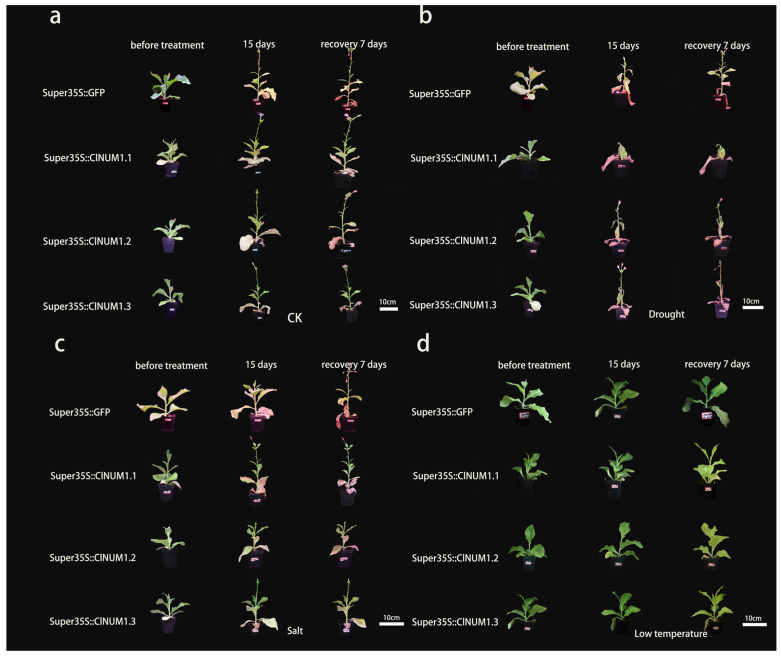
Growth states of mature tobacco seedlings under different treatments. (**a**) Growth state under the control treatment. (**b**) Growth state under drought treatment. (**c**) Growth state under salt treatment. (**d**) Growth state under low-temperature treatment.

In this study, it was found that under the control treatment, transgenic tobacco did not show a significant difference in stem height change after treatment with the same growth rate, but after rewatering, *ClNUM1.1*, *ClNUM1.2*, and *ClNUM1.3* showed significantly slower growth rates compared to CK (Figure 5). 

**Figure 6:**
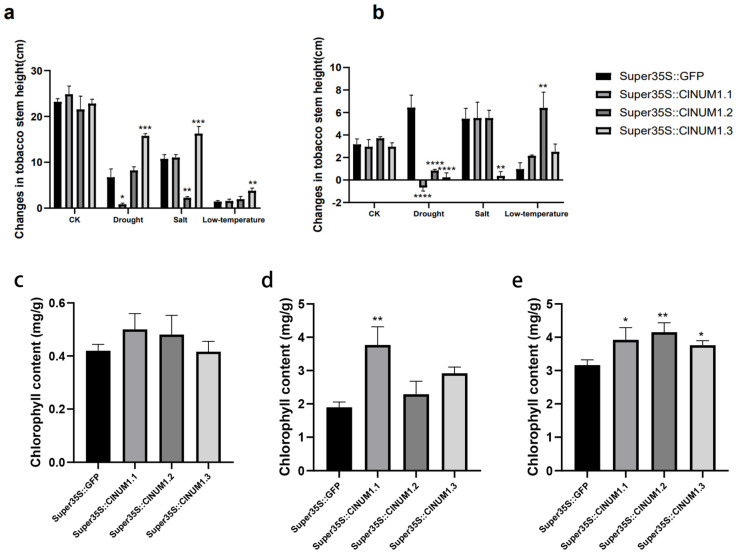
Changes in stem height of mature tobacco seedlings after different treatments and after rehydration and chlorophyll content after treatments. (**a**) Changes in stem height of tobacco after stress treatment. (**b**) Changes in stem height after 7 days of restoration to normal growth conditions under salt, drought, and low-temperature stresses. (**c**) Chlorophyll content after drought treatment. (**d**) Chlorophyll content after salt treatment. (**e**) Chlorophyll content after low-temperature treatment. All experiments were set up with three or more biological replicates (independent sample *t*-test; * *p* < 0.05, ** *p* < 0.01, *** *p* < 0.001, **** *p* < 0.0001).

Under drought treatment, the stem height of *ClNUM1.2* and *ClNUM1.3* increased by 1.5 cm and 9.1 cm compared with that of CK, but the growth recovery rate of *ClNUM1* transgenic tobacco was significantly slower than that of CK after rehydration; the stem height of *ClNUM1.1* exhibited a slower growth rate than that of CK after both treatment and rehydration. Under salt treatment, the stem height of *ClNUM1.1* increased by 0.27 cm and 0.05 cm after both treatment and rehydration compared with that of CK; the stem height of *ClNUM1.2* grew at a slower rate than that of CK after treatment but recovered very quickly after rehydration; and the average stem height of *ClNUM1.3* was 5.5 cm higher than that of CK after treatment but recovered very slowly after rehydration. The average stalk heights of *ClNUM1.1*, *ClNUM1.2*, and *ClNUM1.3* were 0.13 cm, 0.54 cm, and 1.1 cm higher than that of CK under the low-temperature treatment, and they recovered better than CK after rewatering (Figure 6a,b).

Under drought treatment, the chlorophyll content in *ClNUM1* transgenic tobacco was not significantly different from that in CK; under salt treatment, the chlorophyll content of *ClNUM1.1* transgenic tobacco was twice as high as that of CK, with a significant difference, and the chlorophyll content of *ClNUM1.2* and *ClNUM1.3* transgenic tobacco was 1.2 and 1.5 times as high as that of CK, with an increase from CK but no significant difference; the chlorophyll contents of *ClNUM1.1*, *ClNUM1.2,* and *ClNUM1.3* transgenic tobacco were 1.24, 1.31, and 1.2 times higher than those of CK, respectively, under low-temperature treatment, all of which were significant differences (Figure 6c–e).

The test results showed that *ClNUM1.1* transgenic tobacco had strong salt and low-temperature tolerance at the mature seedling stage; *ClNUM1.2* transgenic tobacco had strong low-temperature tolerance; and *ClNUM1.3* transgenic tobacco had strong low-temperature tolerance.

### 3.5. Anatomical Analysis of Transgenic Tobacco Stalks

**Figure 7:**
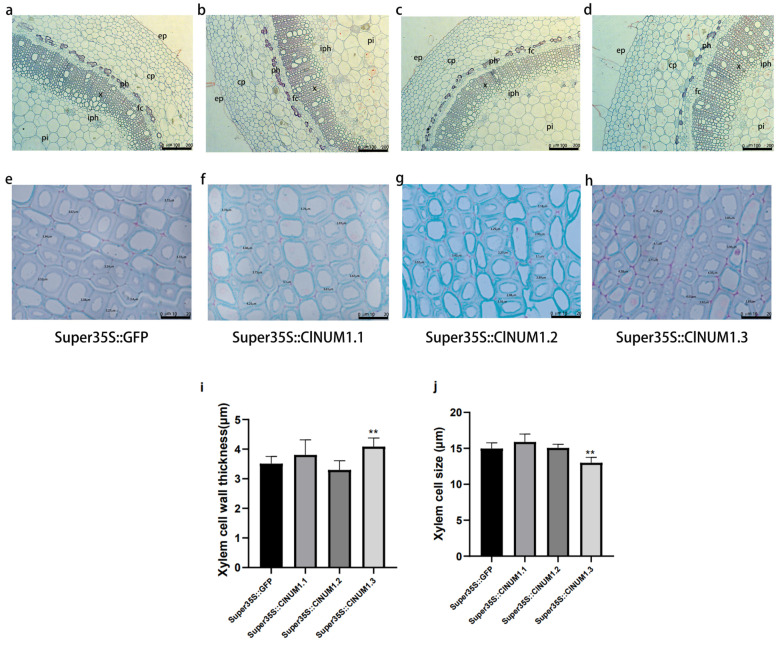
Paraffin sections of transgenic tobacco stems as well as xylem cell wall thickness and xylem cell size of different genotypes of tobacco. (**a**–**h**) Stem cross-sections of CK, *ClNUM1.1*, *ClNUM1.2*, and *ClNUM1.3* transgenic tobacco under normal growth conditions. (**i**) Xylem cell wall thickness of different genotypes of tobacco. (**j**) Xylem cell size of different genotypes of tobacco. Twenty cell sizes and cell wall thicknesses were measured for each transgenic plant. ep = epidermis, cp = cortical parenchyma, ph = phloem, iph = inner phloem, fc = osteocortex, x = xylem, pi = pith. (independent sample *t*-test; ** *p* < 0.01.)

Paraffin sections were taken from one-third of 12-week-old transgenic tobacco stalks and stained with Senka solid green, and the structures of the epidermis, cortex, bast, xylem, and pith could be observed under a light microscope (Figure 7a–h). Both saffron and solid green stain the cell walls of plants; saffron stains the lignified and corky cell walls as well as the ducts of plants red, while solid green stains the cellulose cell walls as well as the sieve tubes of plants green. We measured cell wall thickness and cell size of twenty each in the xylem of transgenic tobacco stalks (Figure 7i,j). Through observation, we found that the cell wall thickness of *ClNUM1.1* and *ClNUM1.3* transgenic tobacco increased compared to that of CK by 1.08-fold and 1.16-fold, respectively, and the cell size also increased compared to that of CK by 1.06 and 1.11 times, respectively. The cell wall thickness and cell size of *ClNUM1.3* transgenic tobacco were significantly different; the cell wall thickness and cell size of *ClNUM1.2* transgenic tobacco were both reduced compared to those of CK with no significant differences.

## 4. Discussion

Alternative splicing is an important pathway for eukaryotes to generate significant regulatory and proteomic complexity. Alternative splicing has two main outcomes—proteome diversification and gene expression regulation [36]—and alternative splicing can increase transcriptome variability and complexity. It is considered to be one of the possible sources of large phenotypic differences between species. In the clipping type, alternative splicing of 5′ and 3′ splice sites is performed by selecting either a 5′ splice donor or a 3′ splice acceptor to retain or splice all or part of the exon sequence [37]. In this paper, the splicing variants we obtained were A5SS and A3SS, which enriches the chrysanthemum variable splicing types. In many studies, alternative splicing has been shown to play an important role in the growth and development of plants and animals, as well as in the response to adversity.

The *ClNUM1* gene was discovered by chance during the cloning of the homologous gene *CcNST1* in *C. lavandulifolium*, and a NUMOD3 conserved domain was found in this gene. Since the gene is a new and unknown gene, we named it *ClNUM1*. In addition, during the cloning process, we found three splicing variants of the *ClNUM1* gene, named *ClNUM1.1*, *ClNUM1.2*, and *ClNUM1.3*.

We characterized the functions of the different splice variants by analyzing morphological and physiological indices of seedling and mature lines of transgenic tobacco under abiotic stress treatments. It was found that the three splice variants enhanced the stress resistance of tobacco (seedling and maturity) and promoted plant growth to some extent, which was consistent with the function of the NAC transcription factor family [38,39].

In the seedling stress experiment, we found that the root length and fresh weight of *ClNUM1* transgenic tobacco were not positively correlated under stress conditions, and sometimes the root length increased but fresh weight decreased or vice versa. In the stress experiment at maturity, we found two interesting phenomena: one is that the growth potential of *ClNUM1* transgenic tobacco under stress conditions was significantly higher than that of CK after treatment, but the recovery rate of the growth potential after rewatering was slower than that of CK. The other is that the growth potential of *ClNUM1* transgenic tobacco after treatment was not significantly different from that of CK, but the recovery rate of the growth potential after rewatering significantly differed from that of CK. We speculate that this may be a response of the plant to resist external environmental changes.

Previous studies have found that NST can thicken the secondary cell wall of Populus and Arabidopsis [40,41], and the change of the secondary cell wall can enhance grapevine and Populus resistance to abiotic stress [42,43]. By observing the cross-section of transgenic tobacco, it was found that compared with CK, the secondary cell wall thickness of transgenic tobacco has changed to a certain extent and the resistance to abiotic stress has been enhanced. We speculated that this gene can regulate the change of plant cell wall thickness and thus regulate plant resistance; this is consistent with previous studies. Abiotic stress will affect the chlorophyll metabolism of plants and thus affect the chlorophyll content of plants [44]. The strength of plant photosynthesis can be reflected by measuring the chlorophyll content so as to evaluate the growth status of plants under abiotic stress. Under the treatment, the chlorophyll content of transgenic tobacco was higher than CK, indicating that the growth status of transgenic tobacco was better than CK, and the resistance was higher than CK.

We have made a preliminary validation for *ClNUM1* function in tobacco based on this experiment; however, how *ClNUM1* in *C. lavandulifolium* regulates plant stress resistance more precisely by affecting secondary cell walls remains to be further studied. Subsequent studies on genes related to secondary cell wall synthesis will reveal the regulatory network and resistance mechanism of *ClNUM1* in *C. lavandulifolium* under abiotic stress.

## 5. Conclusions

In this study, by analyzing the amino acid sequences of the three splice variants of the *ClNUM1* gene of *C. lavandulifolium*, we found that *ClNUM1.1* and *ClNUM1.2* had two selective splicing types and *ClNUM1.3* had selective 5′ splicing. The function of the splice variants of the unknown *C. lavandulifolium* gene *ClNUM1* was verified in tobacco. It was found that *ClNUM1.1* enhanced salt and low-temperature tolerance, *ClNUM1.2* enhanced salt and low-temperature tolerance, and *ClNUM1.3* enhanced low-temperature tolerance in tobacco. By observing the paraffin sections, we found that the thickness of the xylem cells was related to the growth rate of the plant, with thick cell walls in fast-growing plants and thin cell walls in slow-growing plants. However, the molecular mechanism underlying the role of the *ClNUM1* gene in secondary cell wall synthesis and the synthesis of secondary cell wall components such as lignin, cellulose, and polysaccharides is not clear, which is something we need to focus on in future studies. In this study, we discovered a new unknown gene, *ClNUM1*, in *C. lavandulifolium* and verified the function of the splice variants of this gene in tobacco, thus providing a new potential gene for resistance breeding in *Chrysanthemum* and enriching the diversity of resistance breeding in *Chrysanthemum*.

## Figures and Tables

**Table 1:** The primer sequence for *CsNST1* gene.

*CsNST1* Primer Sequence	
*NST1*-F	5′-ATGCTGTCTTCTACTTTGTAAGCTC-3′
*NST1*-R	5′-TTAATACCCTGTTTCAGAAAATGGATG-3′

**Table 2:** The primer sequence for *Actin7* gene.

*Actin7* Primer Sequence	
*Actin7*-F	5′-TCCGGCTATGTATGTTGCTATTC-3′
*Actin7*-R	5′-AATCTTCATCAAGGGATCGGTAAG-3′

## Data Availability

The original contributions presented in the study are included in the article/Supplementary Materials, further inquiries can be directed to the corresponding author.

## Supplementary Materials

The following supporting information can be downloaded at: https://www.mdpi.com/article/10.3390/cimb46060314/s1, Figure S1: The figure shows a comparison of the nucleotide sequences of *ClNUM1* with its three spliced variants. Table S1: Mean and SD of root length and fresh weight of *ClNUM1* transgenic tobacco at seedling stage after treatment. Table S2: Mean and SD of stem height change in mature *ClNUM1* transgenic tobacco after treatment and after recovery.
