# Supplementary material for: Functional Validation of Different Alternative Splicing Variants of the Chrysanthemum lavandulifolium ClNUM1 Gene in Tobacco
Source: Curr Issues Mol Biol. 2024 May 25;46(6):5242–56. doi: 10.3390/cimb46060314 (PMC11201747; doi:10.3390/cimb46060314)
Supplement: Supplementary file 1 [file cimb-46-00314-s001.zip › cimb-2979481-supplementary.pdf]

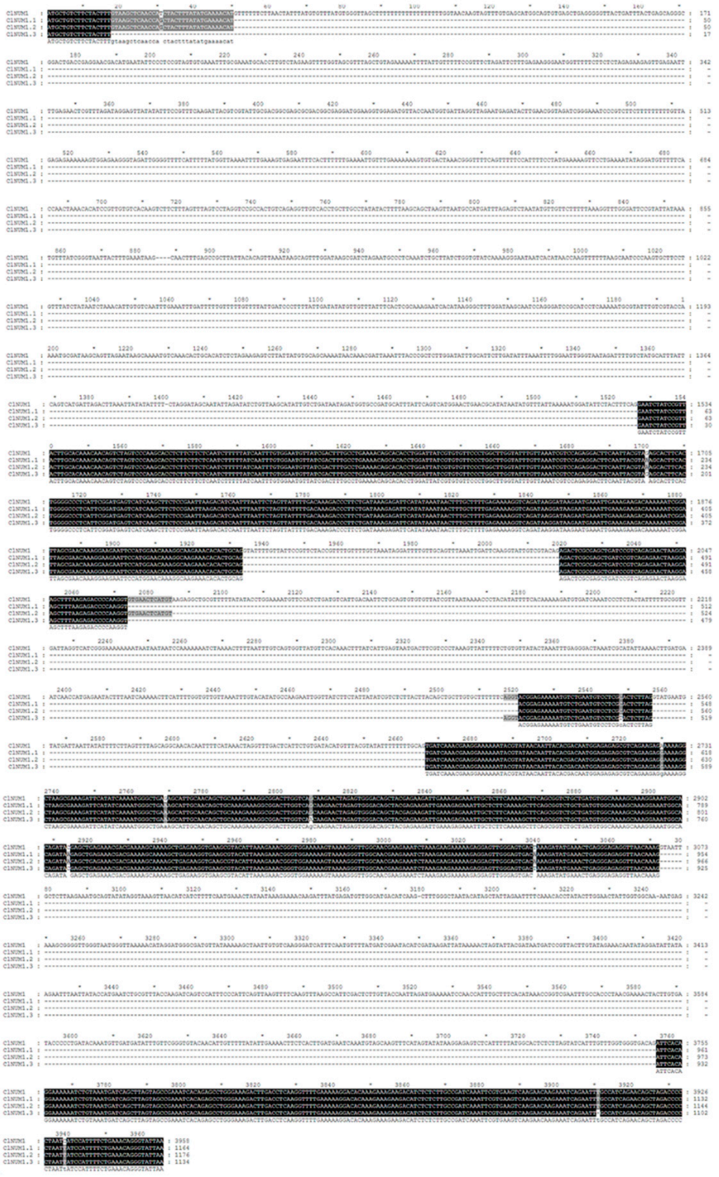

Figure S1. The figure shows a comparison of the nucleotide sequences of *ClNUM1* with its three spliced variants.

Table S1. Mean and SD of root length and fresh weight of *ClNUM1* transgenic tobacco at seedling stage after treatment.

Treatment	Gene name	Mean± SD (root length)	Mean± SD (fresh weight)
CK	Super35S	4.20±0.25	0.46±0.08
	ClNUM1.1	3.90±0.10	0.58±0.03
	ClNUM1.2	4.48±0.19	0.68±0.05
	ClNUM1.3	4.33±0.29	0.43±0.06
ABA	Super35S	1.10±0.12	0.019±0.002
	ClNUM1.1	2.27±0.10	0.02±0.003
	ClNUM1.2	1.46±0.13	0.03±0.002

	ClNUM1.3	1.30±0.08	0.03±0.001
low-temperature treatments	Super35S	4.10±0.30	0.10±0.02
	ClNUM1.1	5.36±0.61	0.36±0.06
	ClNUM1.2	5.55±0.62	0.29±0.04
	ClNUM1.3	4.78±0.48	0.19±0.02
salt treatments	Super35S	4.39±0.36	0.09±0.02
	ClNUM1.1	4.86±0.17	0.42±0.06
	ClNUM1.2	6.10±0.20	0.69±0.017
	ClNUM1.3	4.27±0.21	0.49±0.036

Table S2. Mean and SD of stem height change in mature *ClNUM1* transgenic tobacco after treatment and after recovery.

Treatment	Gene name	Mean± SD (stem height changes after stress treatment)	Mean± SD (stem height changes after recovery)
CK	Super35S	23.2±0.70	3.17±0.49
	ClNUM1.1	24.8±1.82	2.95±0.63
	ClNUM1.2	21.5±2.90	3.70±0.14
	ClNUM1.3	22.8±0.92	2.95±0.35
drought treatments	Super35S	6.73±1.80	6.43±1.15
	ClNUM1.1	0.83±0.29	-0.67±0.31
	ClNUM1.2	8.23±0.81	0.83±0.12
	ClNUM1.3	15.8±0.47	0.23±0.40
low-temperature treatments	Super35S	1.43±0.23	0.97±0.57
	ClNUM1.1	1.57±0.40	2.15±0.07
	ClNUM1.2	1.97±0.60	6.40±1.41
	ClNUM1.3	3.80±0.57	2.50±0.71
salt treatments	Super35S	10.8±0.93	5.45±0.92
	ClNUM1.1	11.1±0.64	5.50±1.41
	ClNUM1.2	2.27±0.23	5.50±0.72
	ClNUM1.3	16.27±1.55	0.37±0.38
